# Indirect Laser-Mediated Halogenation of Graphene: Implications for Hydrogen Evolution Reaction

**DOI:** 10.1021/acsanm.6c00674

**Published:** 2026-05-07

**Authors:** Farheen Khurshid, Jeyavelan Muthu, Jan Plšek, Martin Kalbáč

**Affiliations:** Department of Low-Dimensional Systems, 86875 J. Heyrovsky Institute of Physical Chemistry, Prague 18200, Czech Republic

**Keywords:** graphene, pulsed-laser, defects, covalent functionalization, halogenation

## Abstract

Laser-assisted functionalization has emerged as a versatile route for patterning and chemical modification of two-dimensional (2D) materials, particularly graphene. However, conventional direct laser processing often induces severe photothermal damage, generating lattice defects that compromise charge transport and limit performance in applications such as electrocatalysis, sensing, and optoelectronics. We present an indirect laser-assisted functionalization strategy that prevents lattice defect formation while allowing covalent halogenation of graphene. N-chlorosuccinimide (NCS) and N-bromosuccinimide (NBS) precursors are selectively irradiated with a 455 nm pulsed laser, generating reactive halogen radicals that diffuse toward the graphene surface and form covalent functionalization without direct exposure of the graphene lattice to the laser. Raman and X-ray photoelectron spectroscopy confirm efficient covalent halogenation. The preserved graphene framework maintains high carrier mobility while introducing catalytically active sites, yielding a 2-fold reduction in overpotential and Tafel slope for the hydrogen evolution reaction compared to pristine graphene. This indirect, radical-mediated pathway provides a generalizable framework for laser-driven, defect-controlled functionalization of graphene and other 2D materials.

## Graphical abstract

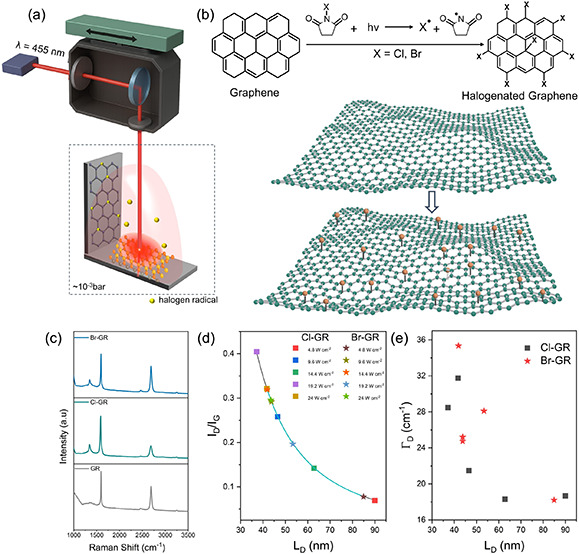

## Introduction

Graphene, with its exceptional electronic, mechanical, and thermal properties, continues to attract significant attention for applications in nanoelectronics, optoelectronics, and sensing technologies.^1–5^ However, its chemical inertness limits direct integration into functional systems, necessitating covalent functionalization strategies to modulate surface reactivity and introduce desirable chemical functionalities.^6,7^ Among various methods, laser-assisted functionalization has emerged as a promising technique due to its spatial selectivity, rapid processing, and compatibility with patterned surface modification.^8–11^ For instance, direct laser irradiation of graphene in the presence of reactive species such as halogens, diazonium salts, or oxygen precursors has been shown to introduce covalent bonds through photoinduced radical reactions effectively.^7,9,12–16^ However, conventional direct irradiation methods often induce significant photothermal damage, leading to lattice disorder and degradation of intrinsic electronic properties.^17–19^ Furthermore, the degree of damage is highly sensitive to laser power, pulse duration, and exposure time, making it difficult to balance between functionalization efficiency and structural preservation.^20,21^

In contrast, defect-selective functionalization enables chemical modification to be confined to preexisting reactive sites such as edges and lattice defects, while preserving the integrity of the basal plane.^22–24^ This nonuniform functionalization is particularly advantageous, as it maintains the extended sp^2^ conjugation required for efficient charge transport while introducing localized active sites for chemical reactivity.^25^ Such a balance is critical for applications in electrocatalysis, sensing, and optoelectronics, where high carrier mobility and controlled surface chemistry must coexist.^26–28^

Herein, we report an indirect laser-induced halogenation of monolayer graphene via photolytic decomposition of N-chlorosuccinimide (NCS) and N-bromosuccinimide (NBS) as radical sources of chlorine and bromine, respectively. In our approach, the laser is focused on a halogen precursor source positioned perpendicular to the graphene surface, preventing direct laser interaction with the graphene while enabling efficient radical generation. The reactive halogen species diffuse toward the graphene surface, where they covalently bind without introducing significant collateral lattice damage. This is corroborated by combined structural and electronic characterizations. The functionalized graphene exhibits enhanced electrocatalytic activity, which can be attributed to the introduction of halogen-based active sites. This indirect laser functionalization strategy offers damage-free functionalization of graphene and other 2D materials.

## Experimental Section

### Graphene Growth and Transfer

Monolayer graphene was synthesized via chemical vapor deposition (CVD) on polycrystalline Cu foil. The Cu substrate was annealed at 1273 K for 20 min under an Ar/H_2_ atmosphere (flow rate = 50 sccm) to increase grain size and surface crystallinity. Methane (CH_4_) was subsequently introduced as the carbon precursor for 30 min at the same temperature. After growth, the system was cooled to room temperature (∼300 K) under continuous Ar/H_2_ flow, yielding predominantly monolayer graphene. For transfer, a PMMA-assisted wet transfer method was employed. The graphene/Cu foil was spin-coated with PMMA (2000 rpm, 30 s) and air-dried. Backside graphene was removed via 10 s oxygen plasma etching. To eliminate chlorine contamination commonly arising from FeCl_3_ etchants, Cu was instead etched using a 0.1 M ammonium persulfate (APS) solution. The released PMMA/graphene film was subsequently rinsed in 0.1 M HCl to remove residual metal ions, followed by multiple washes with deionized (DI) water. The PMMA/graphene film was then transferred onto Si/SiO_2_ substrates, cleaned repeatedly with acetone and methanol, and finally annealed under Ar atmosphere to remove polymer residues and improve substrate adhesion. The duration of each processing step was carefully controlled to ensure reproducibility. Graphene growth was carried out for 30 min following a 20 min annealing step of the Cu substrate, with a subsequent cooling step lasting 1–2 h under an inert atmosphere. The transfer process, including etching, rinsing, and substrate transfer, required approximately 50–60 min in total. Post-transfer thermal annealing was performed at 180 °C for 2 h under Ar to remove residual contaminants. For halogenation, laser irradiation of the precursor was conducted for less than 1 min under a base pressure of ∼10^–3^ mbar.

### Halogenation of Graphene

Halogenation was performed in a custom-built stainless-steel vacuum chamber equipped with a quartz optical window (Figure S1). Approximately 50–100 mg of NCS or NBS was uniformly coated onto a quartz substrate positioned perpendicular to the graphene sample to prevent direct laser exposure. The chamber was evacuated to a base pressure of ∼10^–3^ mbar. A 455 nm pulsed laser (ATOMSTACK MP4 laser engraver, power = 10 W, frequency = 30 kHz, pulse width = 10 ns, and speed = 4000 mm s^–1^) was then directed through the quartz window to irradiate the precursor film, inducing photolytic cleavage of the N–X bond (X = Cl, Br) and generating reactive halogen radicals. These radicals diffused toward the graphene surface and covalently attached to preexisting edge or basal-plane defect sites. After irradiation, the chamber was purged with dry N_2_, and the samples were retrieved. Residual precursors and byproducts were removed by sequential rinsing with acetone and isopropanol, followed by drying under a N_2_ stream. Samples were stored in an inert atmosphere before characterization. The laser functionalization process takes less than a minute.

### Structural and Electronic Characterizations

Raman spectra and mapping were obtained using a WITec Alpha300 spectrometer with a 532 nm excitation laser (1 mW) and a 100× objective. Baseline correction and Lorentzian fitting of D and G bands were performed using WITec Project software. XPS measurements were carried out an Omicron Nanotechnology ESCAProbeP spectrometer, using a monochromatic Al K-Alpha excitation source and on a VG ESCA3MkII spectrometer (base pressure <1 × 10^–9^ mbar) using nonmonochromatic Al Kα radiation. A hemispherical analyzer operated at 20 eV pass energy provided an energy resolution of 1.2 eV (fwhm of Au 4f7/2), with a binding-energy uncertainty of ±0.1 eV.

### Electrical Transport Measurements

Field-effect transistor (FET) devices were fabricated with Au/Cr (50/10 nm) source–drain electrodes patterned onto the graphene channel. A liquid-gate configuration was employed using a 0.5 M Na_2_SO_4_ electrolyte, with a Pt wire serving as the gate electrode. Current–voltage (I–V) characteristics were measured at room temperature using a Keithley 2612B dual-channel source meter controlled via KickStart software.

### Electrochemical Measurements

Electrochemical performance was evaluated using a microdroplet-based three-electrode configuration in 0.5 M H_2_SO_4_. Measurements were conducted with an EmStat4S Potentiostat/Galvanostat (PalmSens BV, The Netherlands) controlled by PSTrace software. A Pt wire and an Ag/AgCl electrode served as counter and reference electrodes, respectively, while a lithographically defined Au pad electrically connected to the graphene acted as the working electrode. Linear sweep voltammetry (LSV) was performed at 10 mV s^–1^ over a potential range of 0.2 to −2 V vs Ag/AgCl. Potentials were converted to the reversible hydrogen electrode (RHE) scale using the following equation.

$$E_{\mathrm{RHE}}{=E}_{\mathrm{Ag}/\mathrm{AgCl}}+0.197V+V_{\mathrm{quasi}}+0.\mathrm{05 pH}$$

Electrochemical impedance spectroscopy (EIS) was conducted over a frequency range of 1 Hz to 0.1 MHz with an AC amplitude of 50 mV at −0.8 V vs Ag/AgCl. Potential-dependent impedance spectra were also collected between 0 and −2 V vs Ag/AgCl at 1 kHz. Carrier densities were extracted from the slopes of the Mott–Schottky plots using the known relative permittivity of pristine and halogenated graphene.

## Results and Discussion

The functionalization of graphene, which includes direct laser exposure, causes severe localized photothermal effects. For instance, absorption of laser photons within the π–π* manifold induces transient heating sufficient to break C–C bonds, generating vacancies and sp^3^-like distortions.^17,29,30^ At higher fluences, local thermal spikes induce ablation, wrinkling, and partial sublimation, resulting in amorphous carbon domains.^19,31^ Under ambient conditions, oxidative reactions further exacerbate these structural changes.^32,33^ To experimentally verify this effect on our samples, the basal plane of CVD-grown graphene was irradiated with a 532 nm laser beam (details provided in the Supporting Information). We observed a power-dependent emergence and growth of the D-band, indicative of defect-activated scattering. Notably, the D-band became detectable even at submilliwatt irradiation levels, reflecting the high sensitivity of the graphene lattice to localized photothermal excitation.

To circumvent photothermal degradation, we implemented an indirect laser-assisted functionalization strategy (Figure 1a). Instead of directly irradiating the graphene, the laser beam was focused onto solid precursors placed near the graphene surface to generate reactive species that enable covalent functionalization (more details are provided in the Supporting Information). This indirect activation route allows for controlled chemical modification without exposing the lattice to destructive photon flux.

**Figure 1:**
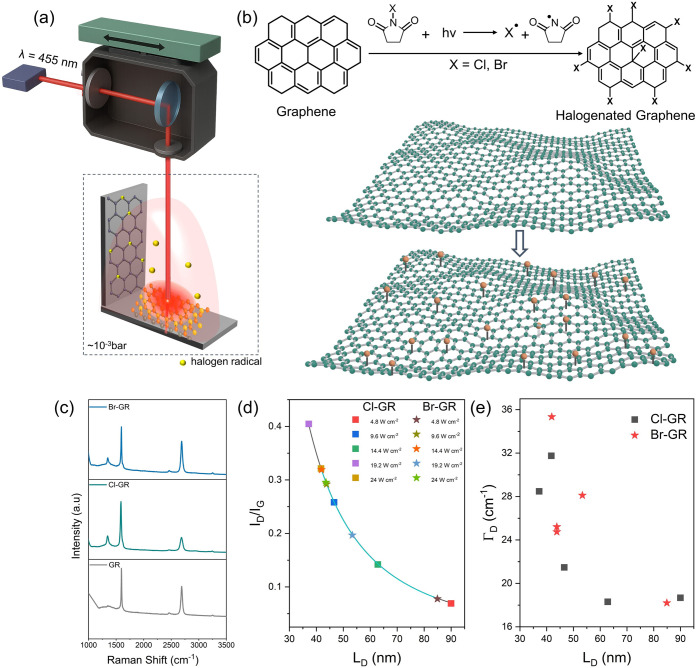
(a) Schematic representation of the indirect laser-assisted functionalization strategy for graphene. In this configuration, the laser beam is directed onto N-chlorosuccinimide (NCS) or N-bromosuccinimide (NBS) precursors, spatially separated from the graphene substrate. (b) Illustration of the laser-induced radical halogenation mechanism: laser excitation of NCS/NBS induces homolytic cleavage of the N–X bond (X = Cl, Br), generating reactive halogen radicals that diffuse toward the graphene surface and covalently bind at edge or activated basal sites. (c) Raman spectra of pristine graphene (GR) and functionalized graphene samples. Quantitative analysis of defect density in halogenated graphene using the Lucchese–Cancado model. (d) Variation of the Raman I_D_/I_G_ ratio as a function of interdefect distance (L_D_) for chlorinated (Cl-GR) and brominated graphene (Br-GR) at different laser powers, along with the fitted Lucchese–Cancado model (solid line). (e) Corresponding evolution of the D-band full width at half-maximum (FWHM, Γ_D_) as a function of L_D_.

N-chlorosuccinimide (NCS) or N-bromosuccinimide (NBS) coated glass substrate was placed inside a custom-built vacuum cell (Figure S1). A CVD-grown graphene film on a Si (coated with 300 nm oxide) substrate was positioned perpendicular to the precursor-coated glass to avoid direct laser exposure and associated heating. A 455 nm pulsed laser was directed onto the precursor film through a quartz window of the vacuum cell (under base pressure 10^–3^ mbar), confining photon absorption to the molecular precursors (Figure 1a). The underlying mechanism can be understood in terms of radical diffusion and reaction kinetics. Upon laser irradiation, N-chlorosuccinimide (NCS) and N-bromosuccinimide (NBS) undergo photolytic cleavage to generate halogen radicals (Cl· and Br·),^34^ which diffuse in vacuum toward the graphene surface (Figure 1b). The subsequent adsorption and reaction of these radicals are dictated by local energy barriers, which are significantly lower at/near defect sites compared to the pristine basal plane. This interpretation is consistent with previous molecular dynamic simulations,^35^ which demonstrate that halogen radicals preferentially bind to defect activated sites due to reduced energy barrier and enhanced local reactivity.^36,37^

The small D-band intensity after functionalization indicates the extended basal plane remains largely undisturbed (Figure 1c), confirming that the indirect laser-assisted functionalization effectively suppresses photothermal defect generation. The I_D_/I_G_ Raman intensity mapping (Figure S2a and b) provides compelling evidence for the site-selective halogenation. To further validate the site-selective nature of the functionalization, graphene was intentionally pretreated with a mild O_2_ plasma (2 s) to introduce more defect sites prior to halogenation.^22^ Raman spectroscopy (Figure S9a) reveals a significant increase in the I_D_/I_G_ ratio from ∼0.13 (pristine) to ∼1.19 after plasma treatment, confirming the generation of defects. Upon subsequent laser-assisted chlorination, the I_D_/I_G_ ratio further increases to ∼2.21. Spatial Raman mapping (Figure S9c–e) shows a consistent enhancement of the I_D_/I_G_ ratio following halogenation across the predefected regions. This progressive increase indicates that the introduced defects serve as preferential binding sites for halogen species.

To quantitatively evaluate the defect density introduced during halogen functionalization, we performed laser power–dependent halogenation, where the laser power governs the generation of reactive radicals. Raman spectroscopy (Figure S7) was then used to monitor the evolution of the defect-activated D-band. The distance between the defects (L_D_) can be determined using eq 1

1 $$L_{D}\left(nm\right)=\left(2.4\times 10^{-10}\right)\times \lambda^{4}{\left(\frac{I_{D}}{I_{G}}\right)}^{-1}$$

The extracted L_D_ values fall within the range of approximately 30–90 nm, depending on the laser fluence, corresponding to a low defect density in the functionalized samples (Figure S7). Such large interdefect distances indicate that the graphene lattice remains largely intact, with only sparse covalent modification sites introduced during the indirect laser-assisted halogenation process. The functionalization is therefore predominantly localized at edge and defect-rich domains, where the local chemical potential and reactivity toward halogen radicals are higher.^36,38^ This observation is consistent with the modest increase in the I_D_/I_G_ ratio (≤0.4) and the preservation of a pronounced 2D-band in the Raman spectra, confirming that the functionalization proceeds without extensive lattice disruption.

The data were further analyzed using the Lucchese–Cancado model,^39^ which correlates the I_D_/I_G_ ratio with the average distance between defects (L_D_). According to this model, the D-band intensity arises from intervalley scattering processes activated within a defect-perturbed region surrounding each sp^3^ site. The relationship between I_D_/I_G_ and L_D_ can therefore be used to determine the spatial distribution of defects in graphene.

2 $$\frac{I_{D}}{I_{G}}=C_{A}\frac{\left(r_{A}^{2}-r_{S}^{2}\right)}{\left(r_{A}^{2}-2r_{S}^{2}\right)}\left[e^{-\pi r_{S}^{2}/L_{D}^{2}}-e^{-\pi \left(r_{A}^{2}-r_{S}^{2}/L_{D}^{2}\right)}\right]$$

Figure 1d shows the experimentally measured I_D_/I_G_ ratio as a function of L_D_ for chlorinated (Cl-GR) and brominated graphene (Br-GR). The experimental data were fitted (solid lines) using the Lucchese–Cancado defect model with the structural disorder radius fixed at r_S_ = 1 nm, consistent with a previous report for covalent functionalization of graphene.^40^ From the fitting, the parameters describing the defect-activated scattering radius were extracted as r_A_ ≈ 4.2 nm for chlorinated graphene, and r_A_ ≈ 4.3 nm for brominated graphene. These values are slightly higher than the previously reported range (3–4 nm).^39–42^ This deviation arises from the intrinsic limitations of applying the Lucchese model in the low defect density regime (L_D_ > 10 nm), where the eq 2 simplifies to $I_{D}/I_{G}=C_{A}\pi (r_{A}^{2}-r_{S}^{2})/L_{D}^{2}$ and becomes less sensitive to the independent determination of individual parameters.

Further insight into the nature of the defects was obtained by analyzing the full width at half-maximum (FWHM) of the D-band as a function of L_D_ (Figure 1e). As the interdefect distance increases, the D-band line width decreases, indicating reduced phonon scattering and improved lattice coherence. Both Cl-GR and Br-GR exhibit similar trends, suggesting that the halogenation mechanism produces comparable defect structures regardless of the halogen species. The observed line widths (≈18–35 cm^–1^) are consistent with isolated sp^3^ defects rather than extended disorder or amorphous carbon formation.

The halogenation of graphene was verified by X-ray photoelectron spectroscopy (XPS). The survey spectrum of pristine graphene (Figure 2a) shows no detectable chlorine signal, which is noteworthy given that chlorine contamination frequently arises from FeCl_3_-based Cu etchants used during graphene transfer.^43^ To eliminate such unintentional doping, graphene was released from the Cu substrate using ammonium persulfate (APS) and subsequently annealed at 300 °C under an inert atmosphere. Following functionalization, clear halogen-related signals emerged. In particular, a Cl 2p peak centered at ∼201 eV (Figure 2a) was observed for chlorinated graphene,^44^ while brominated graphene exhibited characteristic Br 3d (∼70 eV) and Br 3p doublet peaks at 183.5 and 190.5 eV,^45^ confirming successful chlorination and bromination, respectively. High-resolution XPS spectra were further acquired to elucidate the chemical bonding states of the incorporated halogens. The Cl 2p spectrum (Figure 2b) of the chlorinated graphene consists of two principal components at 200.4 and 201.9 eV, which are assigned to covalent C–Cl bonds.^43^ Importantly, the absence of features near ∼198 and ∼206 eV rules out the presence of Cl^–^ or chlorate (ClO_3_^–^) impurities. The high-resolution Br 3p spectrum (Figure 2c) of the brominated graphene exhibits a well-defined spin–orbit doublet. Deconvolution reveals four components corresponding to two Br 3p doublets. The peaks at 183.6 and 190.5 eV are attributed to covalently bonded C–Br species, whereas the lower binding energy components at 181.6 and 188.6 eV are associated with physiosorbed Br^–^ moieties.^46^ We note that the high-resolution C 1s spectra cannot be used as direct evidence of the formation of C–Cl and C–Br bonds due to an overlap of C–Cl and C–Br components with C–O species (see Figure S4a and b).

**Figure 2:**
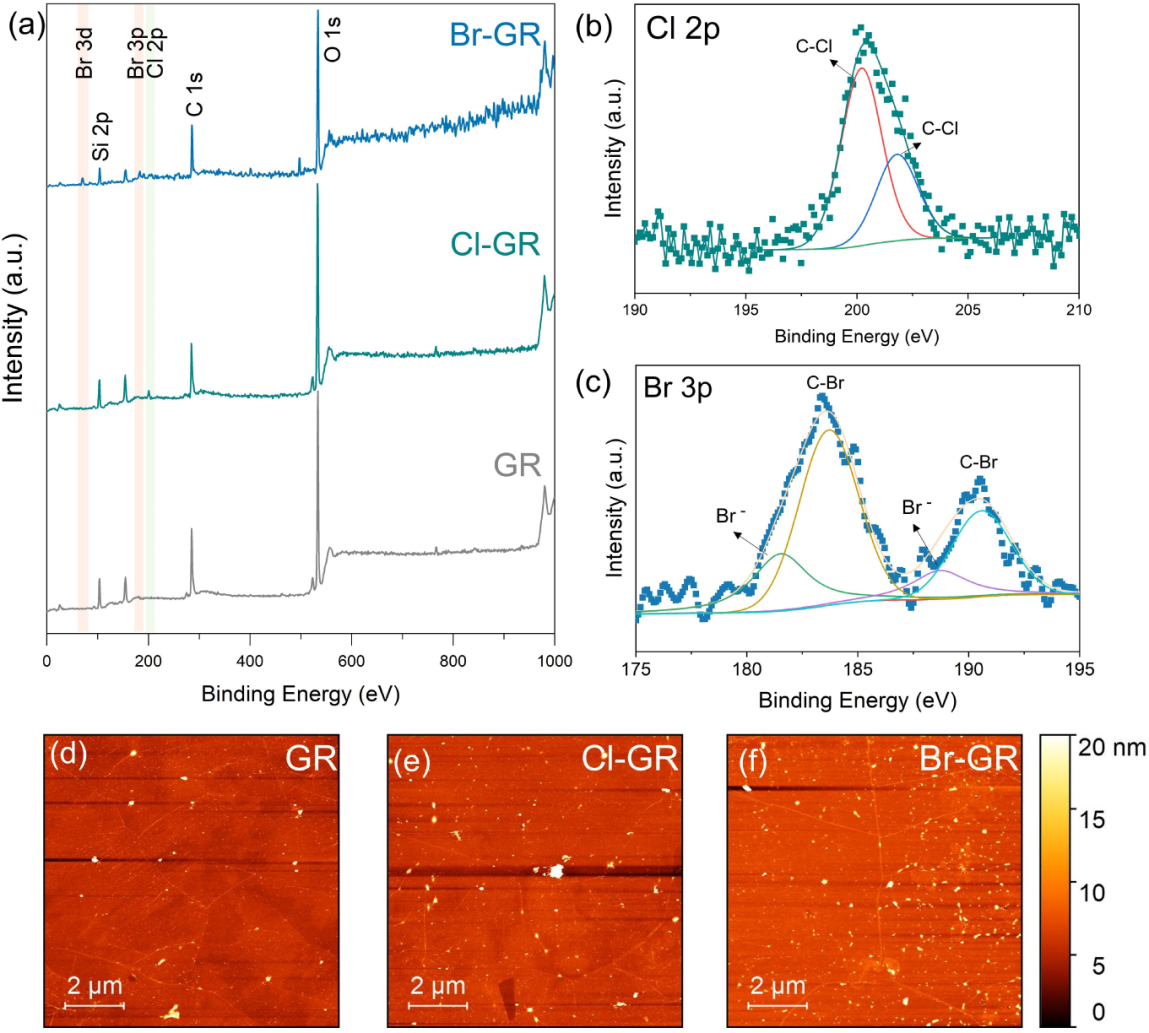
(a) X-ray photoelectron spectroscopy (XPS) survey spectra of GR, Cl-GR, and Br-GR, showing the appearance of characteristic Cl (2p) and Br (3d and 3p) signals after functionalization. High-resolution XPS spectra showing (b) Cl 2p and (c) Br 3p peaks and their corresponding deconvoluted components, confirming the formation of covalent C–Cl and C–Br bonds without detectable inorganic or oxidized contaminants. (d–f) Atomic force microscopy (AFM) images of graphene samples before and after indirect laser-assisted halogenation.

Atomic force microscopy (AFM) was performed to examine the surface morphology before and after halogenation (Figure 2d–f). Chlorinated graphene exhibits a morphology comparable to pristine graphene, indicating minimal surface contamination and preservation of the underlying lattice. In contrast, brominated graphene exhibits discrete surface features, which are likely associated with residual precursor species or weakly bound bromine clusters. XPS analysis reveals the presence of nitrogen signals in both pristine and brominated graphene (Table S1), indicating a baseline contribution from the transfer process or ambient contamination. However, the enhancement of the N signal in Br-GR suggests an additional contribution from NBS-derived residues, consistent with the observed surface features in AFM.

To probe the reversibility of the functionalization, thermal annealing was performed. Raman spectra (Figure S8) show a progressive decrease in D-band intensity and in the I_D_/I_G_ ratio, indicating partial removal of halogen species and a degree of recovery of the sp^2^ lattice. However, the I_D_/I_G_ ratio does not return to that of pristine graphene, indicating that the complete restoration of the sp^2^ network is not achieved, as residual defects or lattice distortions remain after defunctionalization.

To assess the impact of halogenation on the electronic characteristics, field-effect transistor (FET) devices (Figure 3a) were fabricated using both pristine and halogenated graphene as the active channels. Transport measurements (Figure 3b and c) were conducted under a liquid-gated configuration with a fixed channel bias of 100 mV and a gate voltage sweep from −1 to +1 V. Pristine graphene exhibited typical p-type behavior, with a Dirac point at V_g_ ≈ 320 mV, commonly attributed to ambient adsorbates such as O_2_ and H_2_O. Following halogenation, both chlorinated and brominated graphene retained p-type conduction (Figure 3c), accompanied by a slight increase in hole concentration. This behavior is consistent with the electron-withdrawing nature of halogen atoms, which stabilize hole carriers by lowering the Fermi level.^47^ Correspondingly, a shift in the Dirac point was observed, with ΔV_g_ ≈ 90 mV for Cl-GR and ΔV_g_ ≈ 10 mV for Br-GR. These modest shifts indicate that the indirect laser-assisted process introduces controlled doping while preserving the intrinsic electronic characteristics of graphene. In contrast, direct laser functionalization results in significantly higher p-type doping and reduced carrier mobility, which can be attributed to C–C bond scission and the formation of sp^3^-rich domains that irreversibly disrupt the conjugated π-network. These results underscore that indirect laser-assisted halogenation modifies the surface chemistry of graphene while preserving its underlying band structure and carrier transport properties, a key requirement for optoelectronic and catalytic applications.

**Figure 3:**
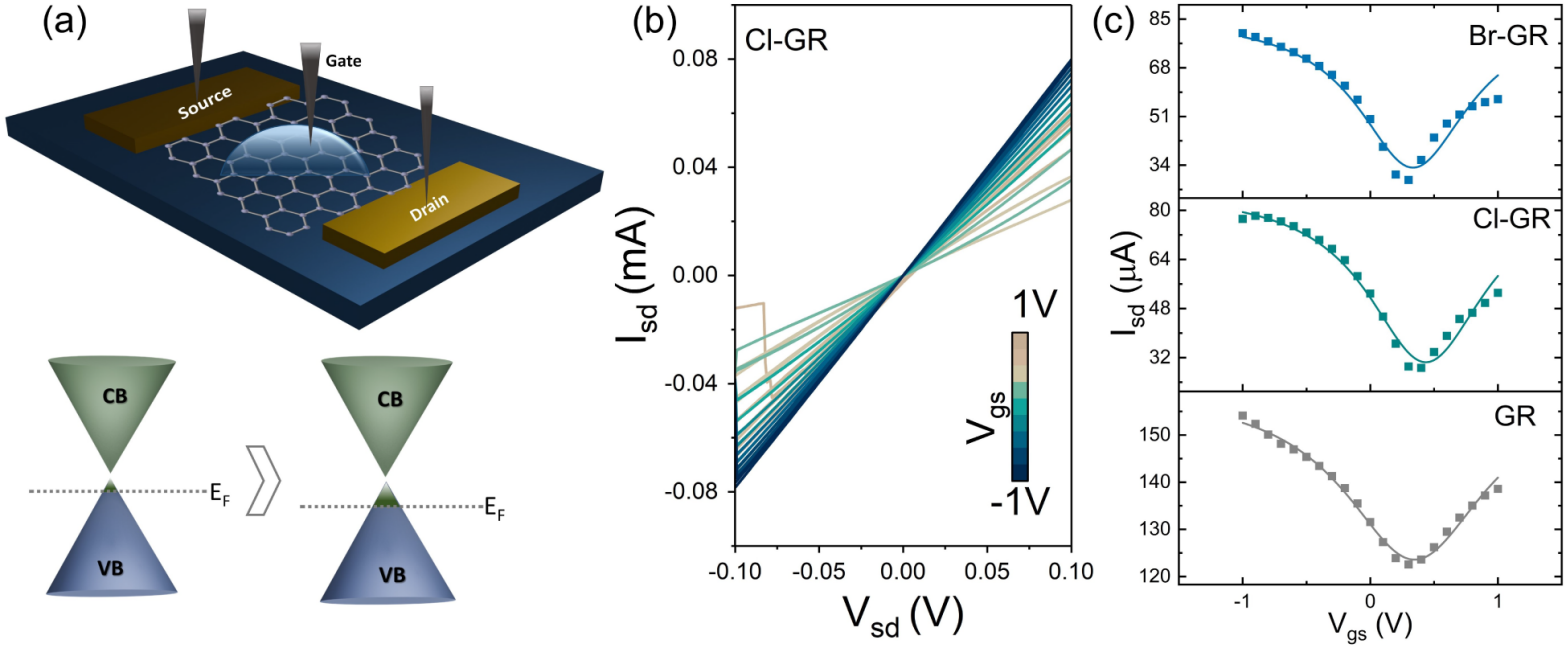
(a) Schematic diagram of the top-gated graphene field-effect transistor (FET) device used for electronic transport measurements and illustration of Fermi level modulation upon halogenation. (b) Representative drain current (I_ds_) versus drain voltage (V_ds_) curves as a function of gate bias for the Cl-GR device, showing preserved ambipolar characteristics. (c) Transfer characteristics (I_ds_–V_gs_) of pristine and halogenated graphene FETs, demonstrating slight positive shifts in the Dirac point consistent with p-type doping induced by electron-withdrawing halogen atoms, while maintaining overall high carrier mobility.

The electrocatalytic performance further reflects these electronic modifications. We have performed electrochemical measurements using a standard three-electrode configuration (Figure 4a). As shown in Figure 4b and c, pristine graphene exhibited limited hydrogen evolution reaction (HER) activity, with an overpotential of 1.16 V and a large Tafel slope of 0.566 V dec^–1^, indicating sluggish kinetics. Chlorinated graphene showed improved activity, reducing the overpotential to 0.92 V and the Tafel slope to 0.272 V dec^–1^, likely due to the introduction of halogen that serves as active sites for hydrogen adsorption.^48^ Brominated graphene demonstrated the highest performance, achieving an overpotential of 0.84 V and a Tafel slope of 0.251 V dec^–1^. The enhanced activity may arise from bromine’s moderate electronegativity and larger atomic radius, which facilitate charge delocalization and efficient catalytic site formation.

**Figure 4:**
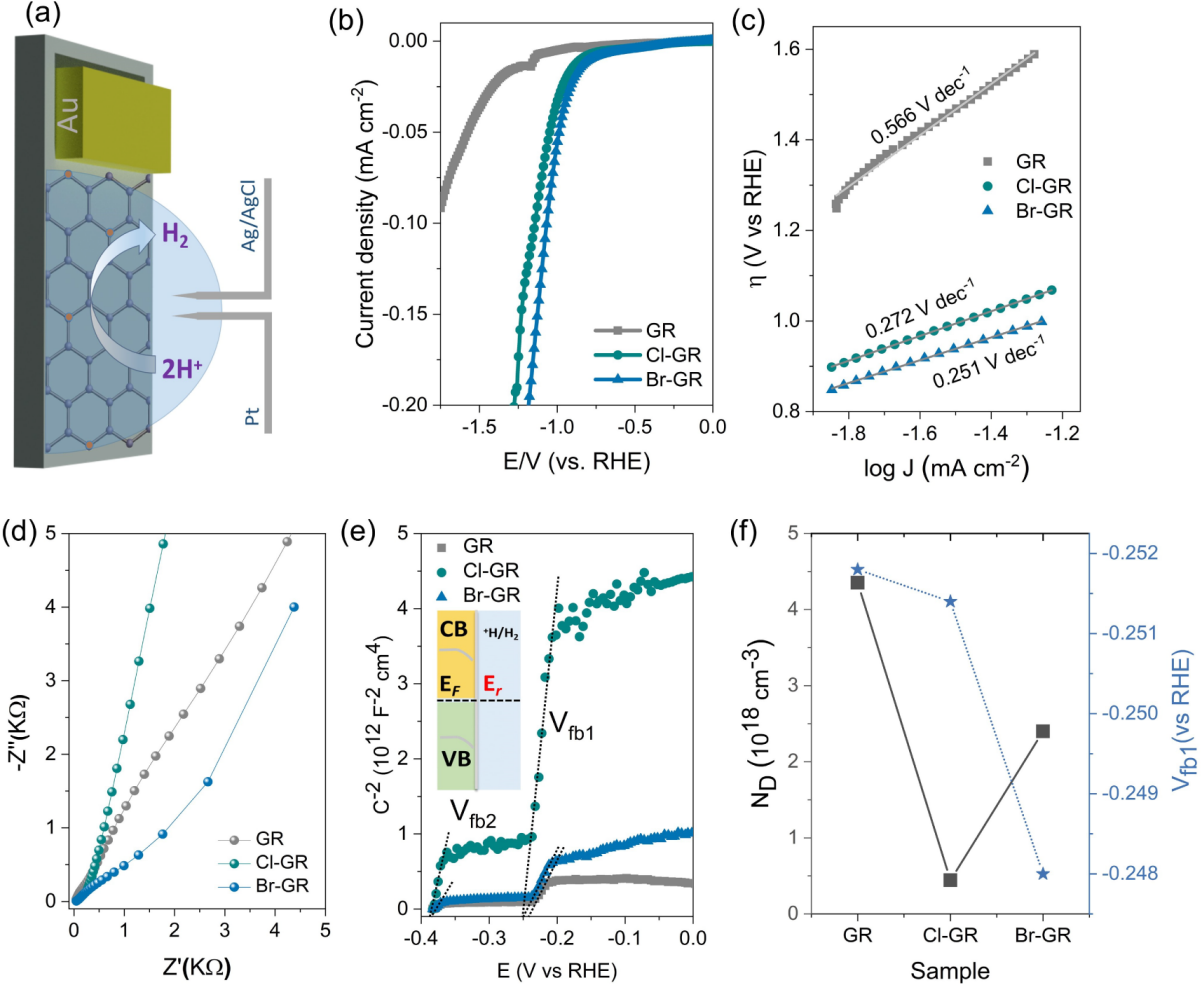
(a) Schematic illustration of the microdroplet three-electrode electrochemical setup used for hydrogen evolution reaction (HER) measurements. (b) Linear sweep voltammetry (LSV) curves of GR, Cl-GR, and Br-GR in 0.5 M H_2_SO_4_, showing significantly enhanced HER activity upon halogen functionalization. (c) Corresponding Tafel plots derived from LSV data, with reduced Tafel slopes for Cl-GR and Br-GR, indicating improved catalytic kinetics. (d) Nyquist plots obtained from electrochemical impedance spectroscopy (EIS) at −0.8 V vs Ag/AgCl, showing reduced charge-transfer resistance (R_ct_) for halogenated samples, particularly Br-GR. (e) Mott–Schottky plots acquired at 1 kHz, illustrating positive shifts in flat-band potentials (V_fb_) upon halogenation and the associated lowering of Fermi levels (inset). (f) Extracted carrier density (N_d_) and corresponding V_fb_ positions for GR, Cl-GR, and Br-GR, confirming p-type doping and the formation of localized electronic states consistent with controlled halogen incorporation.

Electrochemical impedance spectroscopy (Figure 4d) supported these observations. In the Nyquist plot acquired at −0.8 V vs Ag/AgCl, the halogenated graphene samples exhibit smaller semicircles in the high-frequency region compared to pristine graphene, indicating low charge-transfer resistance and enhanced interfacial charge transfer with improved reaction kinetics. Among the functionalized samples, chlorinated graphene shows a higher charge transfer resistance than brominated graphene, which can be attributed to the strong electron-withdrawing nature of chlorine. This effect reduces carrier density and slightly limits charge transport relative to brominated graphene. Mott–Schottky analysis (Figure 4e) revealed a slight positive shift in the flat-band potential (V_fb_) upon halogenation, from −0.20 V for pristine graphene to −0.24 V and −0.21 V for chlorinated and brominated samples, respectively. The apparent charge density (Figure 4f) decreased after halogenation, attributed to partial perturbation of the delocalized π-system due to the incorporation of electron-withdrawing groups.

## Conclusions

In summary, we have developed an indirect laser-assisted functionalization strategy that disentangles chemical modification from laser-induced defect formation in graphene. By selectively irradiating N-chlorosuccinimide and N-bromosuccinimide precursors with a 455 nm pulsed laser, reactive halogen radicals were generated in situ and covalently bound to graphene without direct lattice exposure. This radical-mediated route enables true chemical functionalization while minimizing structural disorder, as confirmed by Raman and X-ray photoelectron spectroscopy. The preserved sp^2^ network maintains high carrier mobility and enhances electrocatalytic performance, leading to a 2-fold reduction in overpotential and Tafel slope for the hydrogen evolution reaction compared to pristine graphene. This indirect approach establishes a general framework for laser-driven, defect-controlled surface chemistry of two-dimensional materials, opening new opportunities for their integration into high-performance optoelectronic and energy conversion devices.

## Supplementary Material



## Notes

Experimental methods and characterization of functionalized graphene samples; indirect laser-assisted functionalization setup; Raman analysis; X-ray photoelectron spectroscopy (XPS) analysis; photothermal and electrical measurements; literature comparison (PDF)
