# Supplementary material for: Indirect Laser-Mediated Halogenation of Graphene: Implications for Hydrogen Evolution Reaction
Source: ACS Appl Nano Mater. 2026 May 7;9(20):9356–63. doi: 10.1021/acsanm.6c00674 (PMC13200163; doi:10.1021/acsanm.6c00674)
Supplement: Supplementary file 1 [file an6c00674_si_001.pdf]

Supporting Information

Indirect Laser-Mediated Halogenation of Graphene: Implications for Hydrogen Evolution Reaction

Farheen Khurshid[a†], Jeyavelan Muthu[a†], Jan Plšek[a], Martin Kalbáč[a*]

[a]Department of Low-Dimensional Systems, J. Heyrovsky Institute of Physical Chemistry, Prague, 18200, Czech Republic

[*]Email: martin.kalbac@jh-inst.cas.cz

[†]Authors contributed equally

1. Indirect Laser-Assisted Functionalization Setup

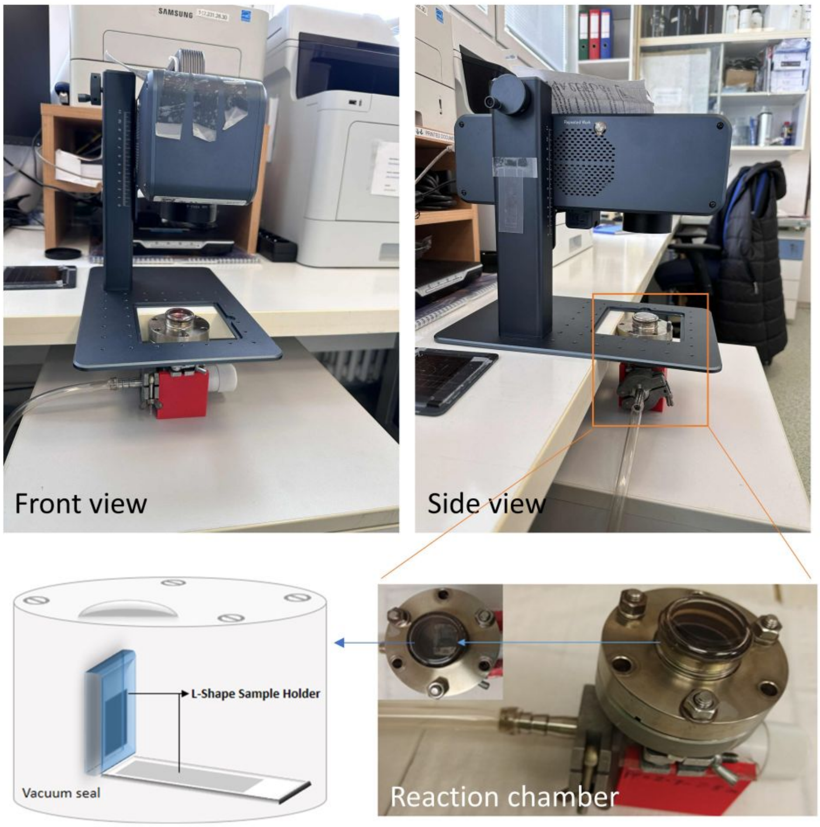

Figure S1. Photographs of the indirect laser-assisted functionalization setup and the custom-built reaction chamber.

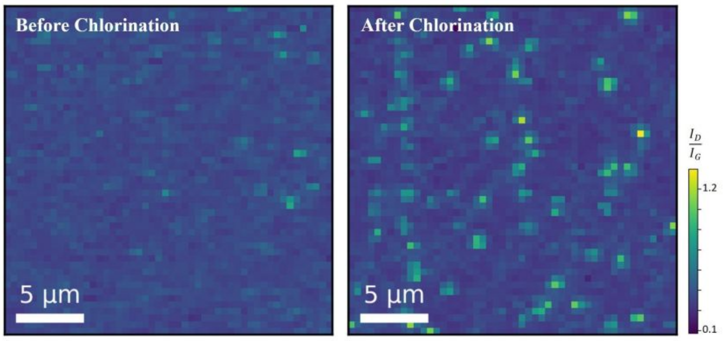

Figure S2. Raman mapping of ID/IG intensity ratios for (a) pristine graphene (Gr) and (b) chlorinated graphene (Cl–Gr).

2. X-Ray Photoelectron Spectroscopy (XPS) Analysis

Quantitative XPS analysis yields Cl/C and Br/C atomic ratios of 5.4% and 5.6%, respectively, confirming effective covalent halogenation of the graphene lattice. Notably, this level of halogen coverage represents a mild and controlled degree of functionalization, sufficient to introduce new chemical functionality while largely preserving the integrity of the sp^2 carbon framework. In the XPS survey spectra of brominated graphene, the Na- signal was also detected. The presence of sodium is attributed to trace contamination originating from the reaction chamber or sample handling environment.

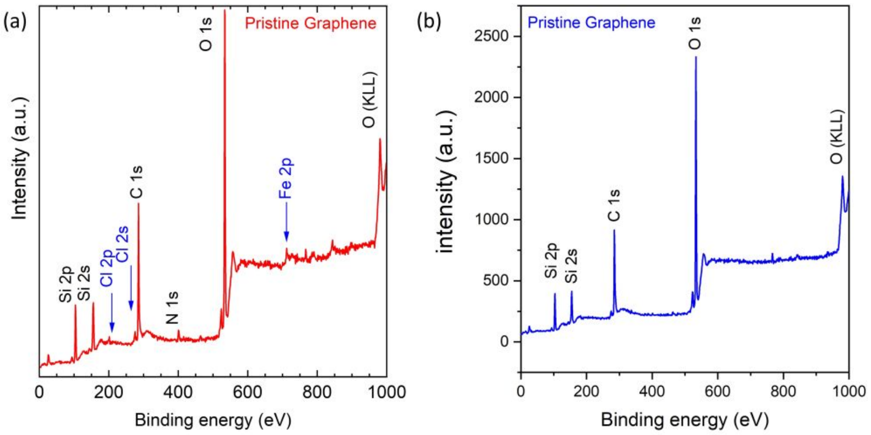

Name	%At Conc
Si 2p	20.81
O 1s	35.74
C 1s	40.03
N 1s	1.91
Fe 2p	0.83
Cl 2p	0.69

Name	%At Conc
Si 2p	19.71
O 1s	39.04
C 1s	40.75
N 1s	0.49
Fe 2p	0.00
Cl 2p	0.00

Figure S3. X-ray photoelectron spectroscopy (XPS) survey spectra of CVD graphene transferred using (a) $FeCl_3$ and (b) ammonium persulfate (APS) solutions, along with the corresponding elemental atomic concentrations extracted from the spectra.

Table S1. Elemental atomic concentrations extracted from the XPS spectra pristine graphene, chlorinated graphene (Cl-GR), and brominated graphene (Br-GR).

Pristine GR

Name	%At Conc
Si 2p	19.71
O 1s	39.04
C 1s	40.75
N 1s	0.49

Cl-GR

Name	%At Conc
Si 2p	20.48
O 1s	43.28
C 1s	34.40
Cl 2p	1.83

Br-GR

Name	%At Conc
Si 2p	13.286
O 1s	40.01
C 1s	35.84
Na 1s	6.083
N	2.768
Br 3p	2.013

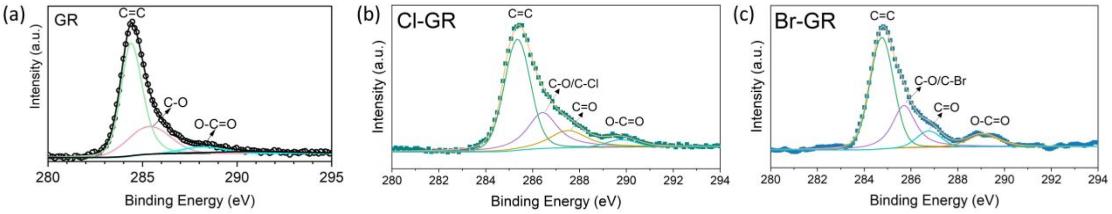

Figure S4. High-resolution spectra showing C 1s peak of (a) pristine, (b) chlorinated and (c) brominated graphene.

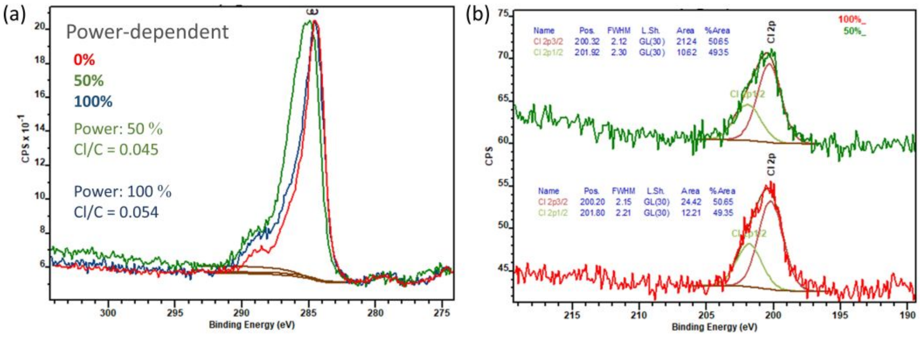

Figure S5. High-resolution XPS (a) C 1s spectra and (b) Cl 2p spectra of chlorinated graphene recorded at different laser power (0, 50, and 100%).

3. Photothermal Impact on Graphene Upon Direct Laser Irradiation

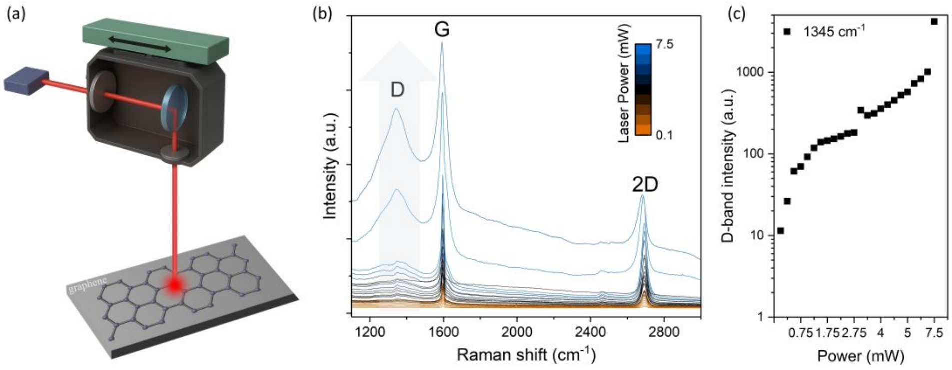

Figure S6. (a) Schematic illustration of direct laser irradiation (λ = 532 nm) on the basal plane of CVD-grown monolayer graphene, showing localized heating and lattice disruption. (b) Power-

dependent Raman spectra of pristine graphene under direct laser exposure at increasing powers (0.1-7.5 mW, indicated by color scale). The progressive emergence and growth of the D band (~1345 cm^{-1}, highlighted by arrows) reflect the onset of defect formation and structural disorder. (c) Evolution of the D-band intensity (I_D) as a function of laser power, revealing a nonlinear increase indicative of photothermal lattice degradation rather than chemical functionalization.

4. Power-Dependent Halogenation

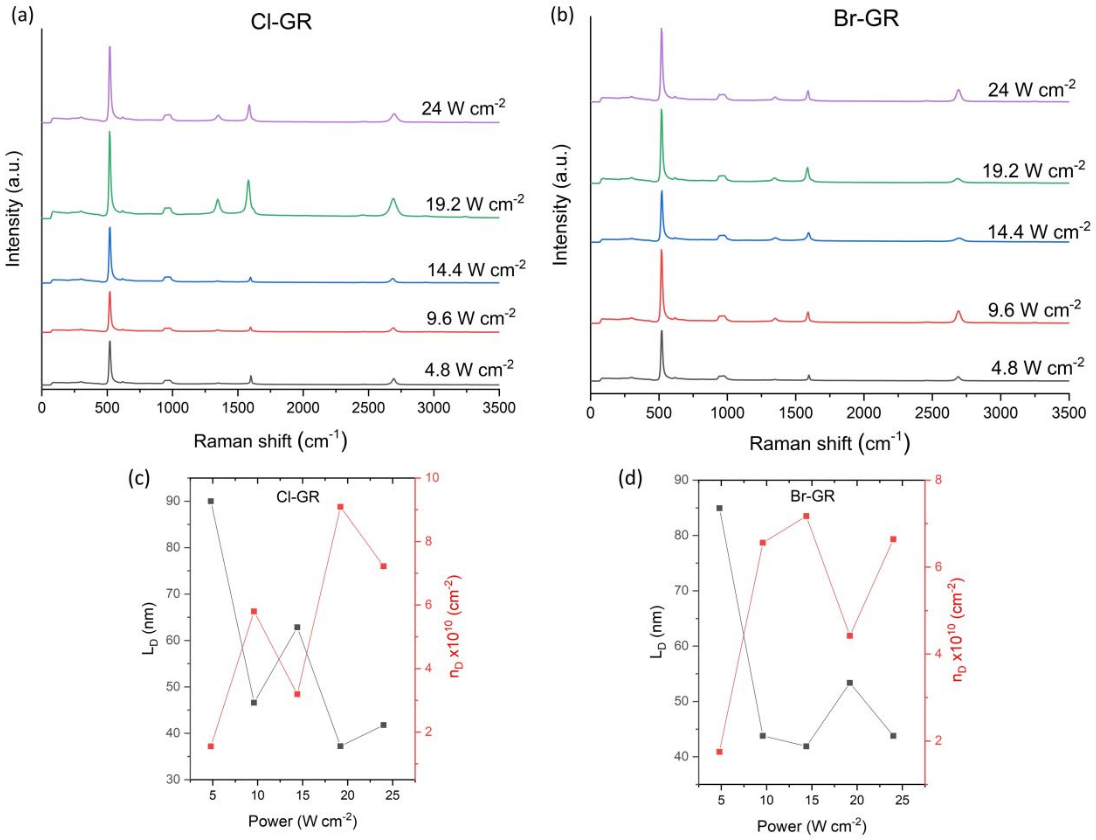

Figure S7. Raman spectra of halogenated graphene as a function of laser power for (a) chlorinated graphene (Cl–GR) and (b) brominated graphene (Br–GR). The spectra show the evolution of the characteristic D, G, and 2D bands with increasing laser power density (4.8–24 W cm^{-2}). (c) and (d) Calculated L_D and n_D as function of laser power for Cl-GR and Br-GR.

5. Reversibility Test

The reversibility of halogen functionalization on graphene was evaluated by monitoring structural changes upon thermal treatment at 298, 423, and 523 K using Raman spectroscopy (Fig. S8). At 298 K, the presence of a pronounced D band confirmed the formation of sp^3-type defects associated with covalent C–X (X = Cl, Br) bonding. Upon annealing at 423 K, a clear attenuation of the D-band intensity was observed, accompanied by a reduction in the I_D/I_G ratio, indicating partial detachment of halogen functionalities and progressive rehybridization of the carbon lattice toward

sp^2 character. Further thermal treatment at 523 K resulted in a more pronounced decrease in the D-band, suggesting substantial reversibility of the functionalization process.

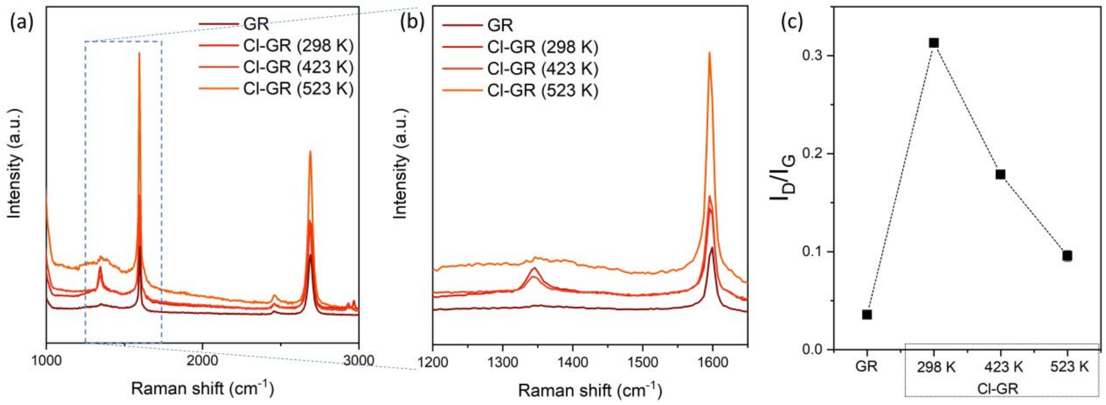

Figure S8. Temperature-induced de-functionalization of graphene monitored by Raman spectroscopy: (a) evolution of Raman spectra upon annealing, (b) enlarged view of the 1200–1650 cm^{-1} region highlighting changes in the D and G bands, and (c) corresponding variation in the I_D/I_G ratio.

6. Defect-Selective Halogenation

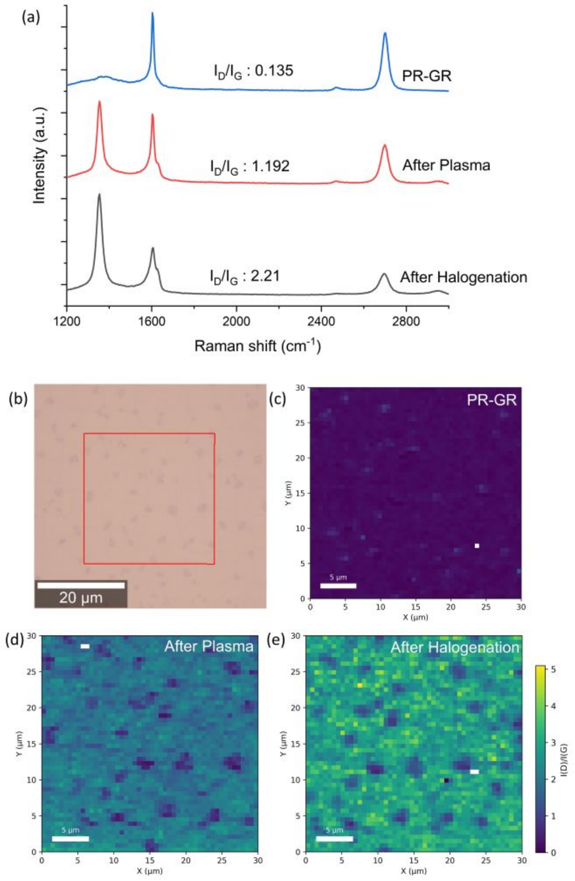

Figure S9. Defect-assisted halogenation of graphene. (a) Raman spectra of pristine graphene, after O_2 plasma treatment, and after subsequent halogenation, showing the evolution of the D and G bands. (b)

Optical microscopy image of the area (rectangle) selected for Raman mapping. (c–e) Corresponding Raman I_D/I_G maps of (c) pristine graphene, (d) plasma-treated graphene, and (e) halogenated graphene, illustrating the progressive increase in defect density and preferential functionalization at defect sites.

7. Topographical and Electrical Characteristics of Functionalized Graphene

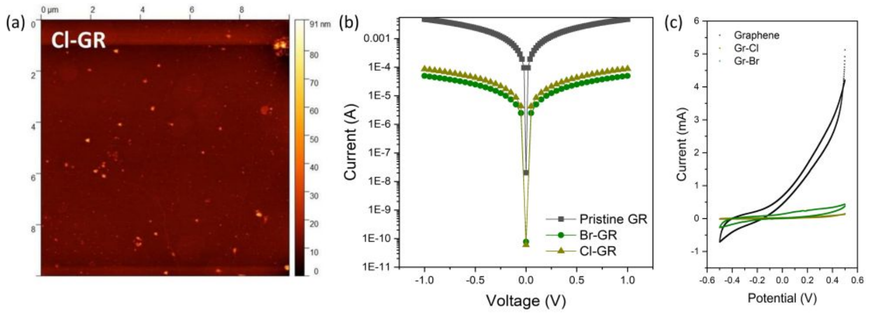

Figure S10. Atomic force microscopy image of chlorinated graphene, (b) two-terminal voltage-current characteristics, and (c) Cyclic voltammetry curves of pristine and functionalized graphene samples.

8. Literature Comparison

Table S2. Literature Comparison of Laser Assisted Functionalization strategies for Graphene.

Strategy	Functional Moiety	Graphene Type	Laser Parameter	Stability	Ref.
Direct-laser functionalization	Phenyl groups	CVD graphene	532 nm, low mW	Reversible	1
Direct-laser functionalization	Perfluorophenyl groups	CVD graphene	UV/visible laser	Irreversible	2
Direct-laser functionalization	Cl/S ($SOCl_2$ doping), O-groups	Multilayer CVD graphene	532 nm, ~40 mJ cm^{-2}	Irreversible	3
Direct-laser functionalization	CF_3 (trifluoromethyl)	CVD monolayer graphene	532 nm, ~mW level	Reversible	4
Direct-laser functionalization	Oxygen functional groups	Vertical graphene nanowalls	ns pulses, ~0.19 J cm^{-2}	Irreversible	5
Direct-laser functionalization	Oxygen functional groups	CVD graphene	515 nm, 30 ps pulses	Irreversible	6
In-direct laser functionalization	Cl, Br	CVD graphene	450 nm pulsed	Reversible	This work

Reference

1. Nagel, T., Gerein, K., Hauke, F., & Hirsch, A. (2025). Laser-Induced Covalent Defunctionalization of Graphene—Precise Patterning and Site-Selective Removal of Functional Groups. Advanced Science, e11481.

2. Nagel, T., Jurkiewicz, L., Hauke, F., & Hirsch, A. (2025). Laser-Initiated Covalent Functionalization of Graphene Using Perfluorophenylazides with Local Addend-Binding Control. physica status solidi (b), 2500348.

3. La Notte, L., Villari, E., Palma, A. L., Sacchetti, A., Giangregorio, M. M., Bruno, G., ... & Reale, A. (2017). Laser-patterned functionalized CVD-graphene as highly transparent conductive electrodes for polymer solar cells. Nanoscale, 9(1), 62-69.

4. Wei, T., Al-Fogra, S., Hauke, F., & Hirsch, A. (2020). Direct laser writing on graphene with unprecedented efficiency of covalent two-dimensional functionalization. Journal of the American Chemical Society, 142(52), 21926-21931.

5. Chaitoglou, S., Klini, A., Papakosta, N., Ma, Y., Amade, R., Loukakos, P., & Bertran-Serra, E. (2024). Processing and functionalization of vertical graphene nanowalls by laser irradiation. The Journal of Physical Chemistry Letters, 15(14), 3779-3784.

6. Bobrinetskiy, I. I., Emelianov, A. V., Otero, N., & Romero, P. M. (2015). Patterned graphene ablation and two-photon functionalization by picosecond laser pulses in ambient conditions. Applied Physics Letters, 107(4).
